# Nuptial gift chemistry reveals convergent evolution correlated with antagonism in mating systems of harvestmen (Arachnida, Opiliones)

**DOI:** 10.1002/ece3.4232

**Published:** 2018-06-22

**Authors:** Penelope C. Kahn, Dennis D. Cao, Mercedes Burns, Sarah L. Boyer

**Affiliations:** ^1^ Biology Department Macalester College St. Paul Minnesota; ^2^ Chemistry Department Macalester College St. Paul Minnesota; ^3^ Biology Department San Diego State University San Diego California; ^4^ Present address: Department of Biological Sciences University of Maryland Baltimore County Baltimore Maryland

**Keywords:** chemical ecology, *Leiobunum*, mating systems, phylogenetics, Sclerosomatidae

## Abstract

Nuptial gifts are material donations given from male to female before or during copulation and are subject to sexual selection in a wide variety of taxa. The harvestman genus *Leiobunum* has emerged as a model system for understanding the evolution of reproductive morphology and behavior, as transitions between solicitous and antagonistic modes of courtship have occurred multiple times within the lineage and are correlated with convergence in genital morphology. We analyzed the free amino acid content of nuptial gift secretions from five species of *Leiobunum* using gas chromatography–mass spectrometry. Multivariate analysis of the free amino acid profiles revealed that, rather than clustering based on phylogenetic relationships, nuptial gift chemical composition was better predicted by genital morphology and behavior, suggesting that convergent evolution has acted on the chemical composition of the nuptial gift. In addition, we found that, species with solicitous courtship produce gifts consisting of a 19% larger proportion of essential amino acids as compared to those with more antagonistic courtship interactions. This work represents the first comparative study of nuptial gift chemistry within a phylogenetic framework in any animal group and as such contributes to our understanding of the evolution of reproductive diversity and the participant role of nuptial gift chemistry in mating system transitions.

## 1 INTRODUCTION

Nuptial gifts are transferred from male to female before or during copulation and may come in many forms, including food or prey items, glandular secretions, and chemical cargo such as minerals or toxic defense (Gwynne, 2008; Lewis & South, 2012; Lewis et al., 2014). Females may derive direct benefits from the gift, as in the case of blister beetles in which males transfer a store of cantharidin toxin to the female which she then apportions to the eggs as chemical defense (Eisner et al., 1996). Males, too, may experience fitness benefits as a result of nuptial gift‐giving; for example, the mating success of the male great gray shrike increases with the quality of the prey item offered (Tryjanowski & Hromada, 2005). There has been considerable effort to document the behavioral and physiological interactions between male gift‐givers and their female recipients and to determine the function of these interactions. Two nonmutually exclusive hypotheses have been proposed: (a) *The paternal investment hypothesis* states that males should provide a gift containing some nutritional or other benefit that improves the female condition, thereby increasing the number or quality of offspring, and (b) *the mating effort hypothesis* proposes that donations support insemination by acting as a sensory trap, preventing the female from terminating copulation, or by prolonging the female remating interval, reducing sperm competition (reviewed by Vahed, 1998 and Gwynne, 2008).

Nuptial feeding has been documented in many arthropod mating systems (reviewed by Vahed, 1998 and Gwynne, 2008). Perhaps due to its greater burden of proof, the paternal investment hypothesis has rarely been invoked, but the mating effort hypothesis has been well supported in several species of cricket (Gershman, Mitchell, Sakaluk, & Hunt, 2012; Gordon, Gershman, & Sakaluk, 2012; Sakaluk, 2000; Warwick, Vahed, Raubenheimer, & Simpson, 2009; Wedell, Tregenza, & Simmons, 2008). Cricket species which participate in nuptial feeding transfer a spermatophore which is composed of two parts: the sperm‐filled ampulla and a gelatinous bulb termed the spermatophylax. Both parts are eaten by the female: first the spermatophylax which covers the ampulla and then the ampulla which remains attached to the female reproductive tract while she is eating the spermatophylax (Warwick et al., 2009). It has been hypothesized that the spermatophylax in this cricket acts as a sensory trap through the use of free amino acids, which have been shown to have a phagostimulatory effect in insects (Calatayud et al., 2002) and allow the male to transfer sperm for longer periods of time (Warwick et al., 2009). Nuptial feeding has also been documented in the spider species *Pisaura mirabilis* (Stålhandske, 2001, 2002) and *Paratrechalea ornata* (Albo & Costa, 2010). The gifts in these systems are composed of prey items wrapped in silk; males present these gifts to females to increase their chance of mating, but the gifts have no effect on female fecundity or size of eggs, so it appears that these gifts likewise function as sensory traps (Albo & Costa, 2010; Stålhandske, 2001, 2002). While we have some knowledge of the biology of nuptial gifts in spiders, when it comes to the arachnid order Opiliones, commonly known as harvestmen, nuptial gifts remain entirely unexplored territory.

**Figure 1:**
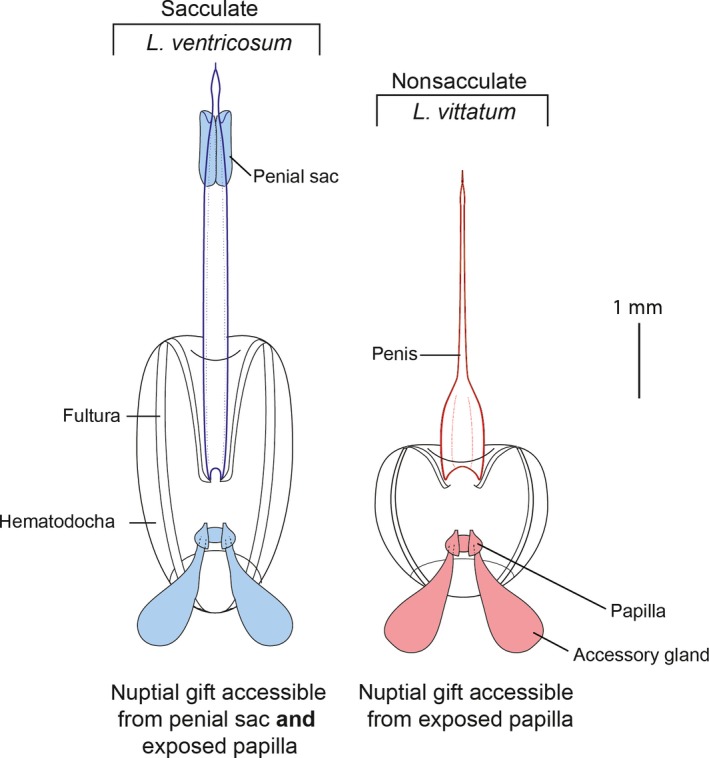
Dorsal views of the two genital morphotypes that occur within *Leiobunum*: sacculate and nonsacculate. Sacculate species bear a pair of subterminal cuticular sacs on the distal penis. Internally, papilla of nuptial gift‐producing accessory glands fill penial sacs. Penes are everted with cuticular fulturae, which support the membranous hematodocha that contains the accessory glands. Upon eversion, papilla are externalized and nuptial gift may be secreted over the hematodocha. Nonsacculate species lack penial sacs, but retain accessory glands

North American leiobunine harvestmen (Opiliones: Sclerosomatidae: Leiobuninae), often colloquially referred to as daddy‐longlegs, offer a compelling system for the study of nuptial gifts. These understudied arachnids participate in nuptial feeding via endogenous glandular secretions, but currently, there is no published research on the chemical composition or effects of the secreted substance on the female. Indeed, very little information has been published on reproductive biology in these animals generally; within the genus *Leiobunum,* the mating behavior of only one species, *L. vittatum,* has been studied in detail (Fowler‐Finn, Triana, & Miller, 2014; Macías‐Ordóñez, 1997). While behavior associated with leiobunine reproduction is largely unexplored territory, the reproductive anatomy in this group has been the topic of several recent phylogenetic and biomechanical studies. Over the last 5 years, Burns, Shultz, and collaborators have documented the evolution of genitalia in this group, uncovering patterns of correlated evolution in male and female genital anatomy strongly suggestive of multiple evolutionary shifts from less antagonistic to more antagonistic interactions between males and females (Burns, Hedin, & Shultz, 2012, 2013; Burns & Shultz, 2015, 2016). Specifically, Burns et al. (2012) identified two broad categories of male leiobunine genital morphology: sacculate and nonsacculate. Sacculate species have a bilateral pair of cuticular sacs on the distal penis, which contain nuptial secretions produced in accessory glands (Figure 1). To initiate mating, the sacculate male engages in a face‐to‐face embrace, locking his pedipalps behind her second pair of coxae, and transfers a primary nuptial gift directly to the female's mouth. He then quickly withdraws and reorients the distal penis to the opening of the female's pregenital chamber. Copulation begins when the female relaxes her genital operculum and the male inserts the glans into the pregenital chamber. During this time, nuptial gift is issued from the accessory glands, the papillae of which are externalized when the penis is everted (Figure 1). This constitutes a secondary gift the female may gather as copulation proceeds. In nonsacculate species, mating begins in a similar way, but the primary nuptial gift transfer is omitted because penial sacs are not present (Burns et al., 2012). Therefore, any acquisition of nuptial gift by the female occurs during clasping and manipulation by the male.

**Figure 2:**
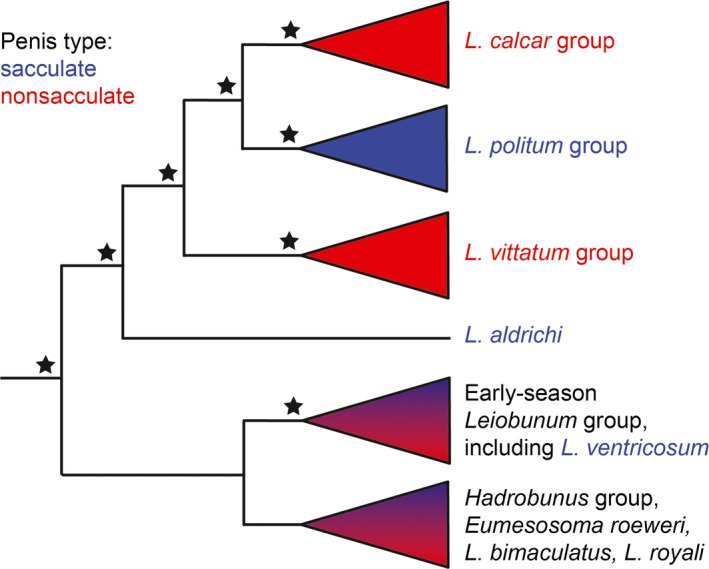
Simplified Bayesian phylogeny of the eastern North American leiobunine harvestmen illustrating evolutionary transitions between sacculate (blue) and nonsacculate (red) genital morphotypes. Names of species used in this study are presented in color. Triangles represent species complexes within which named species are nonmonophyletic. Stars indicate relationships well supported in molecular phylogenetic analyses, that is, 100% support under at least two of three optimality criteria: Bayesian posterior probability, maximum likelihood, parsimony. After Burns et al. (2012, 2013)

With few exceptions, females of nonsacculate species have sclerotized pregenital barriers, presumably to aid in resistance against unwanted advances (Burns et al., 2013). Similar correlations exist between nonsacculate condition and the development of specialized male pedipalps hypothesized to improve grasping of the female during the mating embrace (Burns et al., 2013). Burns and Shultz found that the nonsacculate condition correlated strongly with increases in female pregenital closing force, penis length, and muscle forces associated with deformation of the penis that could achieve a crowbar‐like prying motion against the female pregenital opening (Burns & Shultz, 2015). Moreover, penes of nonsacculate species may transmit and resist higher levels of mechanical force (e.g., bending) when interacting with the female's pregenital opening than the sacculate penes, suggesting that there has been selection for more durable penes in sexually antagonistic species (Burns & Shultz, 2016). Taken together, these syndromic morphologies in the eastern North American leiobunine harvestmen suggest an evolutionary transition in mating system has taken place—with a solicitous courtship style marked by greater accessibility to nuptial gift supplanted by a more antagonistic mating system defined by more restricted access to nuptial gift (Figure 2).

We set out to compare the chemical profiles of the nuptial gifts of five closely related harvestmen species representing the major evolutionary lineages within *Leiobunum* and both the sacculate condition (*L. aldrichi, L. politum, L. ventricosum*) and the nonsacculate condition (*L. calcar, L. vittatum*) in order to identify how compositional differences varied with genital morphotypes. Whether the nuptial gift provides females with a direct benefit (as in the paternal investment hypothesis) or acts as a sensory trap (as in the mating effort hypothesis), we would expect to see changes in the chemical composition of the gift associated with evolutionary transitions in mating strategy. Therefore, our expectation was that we would find significant compositional differences in the nuptial gift correlated with the sacculate and nonsacculate genital morphotypes (Figure 2). We extracted the free amino acids from the accessory glands of 67 individuals and analyzed them using gas chromatography–mass spectrometry (GC‐MS). We used multivariate statistics, including an approach that accounts for data variance due to phylogenetic relatedness, to test for an association between the free amino acid profile of nuptial gift and genital morphology—and therefore level of sexual antagonism. We found evidence of evolutionary convergence in nuptial gift composition correlated with male genital phenotype. Our results set the stage for work that could identify the role of nuptial gift in mating interactions and fecundity in harvestmen.

## 2 MATERIALS AND METHODS

### 2.1 Specimen collection and preservation

Our taxonomic sampling included five species representing most major lineages of leiobunine harvestmen (Figure 2). Sixty‐seven males were collected, including five *L. politum*, fourteen *L. aldrichi*, eighteen *L. calcar*, seventeen *L. ventricosum*, and thirteen *L. vittatum*. All individuals were hand‐collected from one of two locations: Macalester College's Katharine Ordway Natural History Study Area in Inver Grove Heights, MN (June 1 through August 5, 2016; 56 specimens), or Emmenegger Nature Park in St. Louis, MO (June 13, 2016). They were either immediately preserved or housed at Macalester College for use in the mating trials (12 individuals) of another project, after which they were preserved in either 75% or 95% ethanol and stored in the refrigerator to prevent degradation. See Table 1 for summary collection information.

**Table 1:** Specimen information

Species	No. of specimens	Collection locality	Collection date	Preservation	Used in mating trials?
*Leiobunum aldrichi*	4	O	6/01/2016	95%	N
	9	O	8/05/2016	95%	N
	1	O	6/01/2016	75%	N
*L. calcar*	1	E	6/13/2016	Unknown	Y
	8	E	6/13/2016	Unknown	N
	1	O	6/01/2016	Unknown	N
	3	O	6/14/2016	Unknown	N
	1	O	6/14/2016	95%	Y
	1	O	6/14/2016	75%	N
	3	O	6/14/2016	95%	N
*L. politum*	1	O	7/05/2016	95%	N
	1	O	7/06/2016	95%	N
	1	O	8/05/2016	95%	N
	2	O	6/29/2016	75%	N
*L. ventricosum*	1	O	6/03/2016	75%	Y
	4	O	6/03/2016	75%	N
	2	E	6/13/2016	75%	N
	7	O	6/14/2016	75%	Y
	3	O	6/14/2016	75%	N
*L. vittatum*	1	O	6/01/2016	95%	N
	1	O	6/14/2016	95%	N
	13	O	8/05/2016	95%	N
Total	67	Ordway: 56		95%: 33	Used in trials: 10
		Emmenegger: 11		75%: 21	
				Unknown: 13	

### 2.2 Dissection

Preserved specimens were placed ventral side up in a clean, dry petri dish. The genital operculum was cut at the lateral sulci, and the inverted genital structure (i.e., penis, glands, muscles/sheath; Figure 1) was dissected and placed in another clean, dry petri dish and allowed to dry for 5–10 s. With a plastic 2‐ml FALCON dropper, 4–5 drops of deionized water were dropped onto the structure. For sacculate penes, any content in the sacs was scraped out and crushed in the water until it dissolved. Accessory glands, found immediately dorsal and deep to the distal penis and typically identified by gland papillae, were removed and combined with the sac content. For nonsacculate penes, only the accessory glands were extracted and prepared. Following dissolution of the sac/gland material, a clean dropper was used to transfer all of the samples to an Eppendorf 1.5‐ml FLEX tube. The samples were stored at −15°C until extraction.

### 2.3 Free amino acid extraction

We adapted the extraction methods of Warwick et al. (2009), using an anion‐exchange resin to separate the free amino acids from proteins and other macromolecules in the harvestman nuptial gift samples. Frozen samples were thawed and 1 ml Milli‐Q water was added. Samples were heated to 100°C for 5 min using a sand bath in order to dissolve all water‐soluble contents completely. The entire volume was passed through an anion‐exchange column (0.6 g Dowex 1X8‐200 in a glass pipette with a Kimwipe plug) into a 5‐ml glass vial. The column was subsequently washed with 2 ml Milli‐Q water, then 1 ml of 1 M ammonium hydroxide into the same glass vial. The samples were stored at −15°C until they were lyophilized. To prepare for lyophilization, the caps were removed from the vials and a layer of Kimwipe was secured to the opening with a small rubber band. The samples were then flash‐frozen in a dry ice/acetone bath. The frozen samples were lyophilized for 24 hr at −40°C under reduced pressure (<0.1 mmHg), after which they were stored at −15°C until derivatization.

### 2.4 Amino acid derivatization

In order for GC‐MS to detect a sample, the analyte molecules must be sufficiently volatile enough to be ionized in the gas phase. The volatility of the free amino acids was improved by derivatization following methods from Stenerson (2011). 100 μl of a derivatization mixture (1:1 ratio of *N*‐*tert*‐butyldimethylsilyl‐*N*‐methyltrifluoroacetamide (MTBSTFA) and acetonitrile) was added to each sample vial after it was thawed. An analytical amino acid standard from Sigma‐Aldrich (AAS18‐5ML) was prepared by adding 10 μl of the standard 100 μl of the derivatization mix. The samples and standard mixture were then heated in an oil bath at 120°C for 30 min, after which they were removed from heat. 1 ml of acetonitrile was added to the sample vial prior to subjecting it to GC‐MS analysis.

### 2.5 Gas chromatography/mass spectrometry analysis

Gas chromatography–mass spectrometry was used to identify and quantify the relative amount of each free amino acid in the harvestman nuptial gift samples. Samples were analyzed within 24 hr of derivatization. Derivatized samples were kept at 4°C until they were analyzed on an Agilent 5973/6890N GC‐MS system with an Agilent 19091S‐433 model column. A high molecular weight method was employed (oven temperature ramped from 100 to 280°C over 20 min). Additional detail is provided in Supporting Information Data S1.

### 2.6 Data analysis

The GC‐MS spectrum of the amino acid analytical standard was used to identify the amino acid peaks, the fragmentation, and retention time of each amino acid. The fragmentation of each peak of the amino acid analytical standard was compared to a GC‐MS spectrum provided by Sigma‐Aldrich (Stenerson, 2011) in order to confirm the identity of each compound. This information was used to classify the peaks derived from each nuptial gift specimen. We calculated the integrals of each amino acid peak and used these data to create an amino acid profile for each individual. Essential amino acid (EAA) proportions were calculated by summing the integrals for all essential amino acids in an individual's profile and dividing by the integral sum of all amino acids for that individual. The amino acids that are considered essential here are the same as in vertebrates, as Davis (1975) demonstrated that this was the case for another arthropod, the yellow mealworm. We recognize that the amino acids that are “essential” in harvestmen could be different, but there is currently no literature on the matter. An ANOVA comparing the EAA contents of both genital morphologies was performed in JMP Pro 13 (SAS Institute Inc., 1987‐2007) and a phylogenetic ANOVA was performed in R (R Core Team, 2016) with the *phytools* package (Molina‐Venegas & Rodríguez, 2017), using 1,000 simulations and a Holm 1979 post hoc test. PCAs investigating the relationship between species, morphology, and chemical profile were performed in JMP.

In order to determine the effect of shared evolutionary history on the variation in the nuptial gift composition, Pagel's lambda value was calculated in R (Pagel, 1999). Pagel's lambda represents an estimate of the covariance between the traits of the clade and their phylogeny (Pagel, 1999). Lambda values range from 0 to 1, where a value approaching 0 means that the phylogeny does not indicate covariance in amino acid profiles following Brownian motion. A value of 1 indicates amino acid covariances are those expected under a standard Brownian motion model (Revell, 2012). Moreover, a lack of phylogenetic signal in nuptial gift composition indicates that phylogenetic comparative and frequentist statistical methods will perform identically. Pagel's lambda was calculated for this dataset using two equations from different packages in R. Two methods for the calculation of lambda were performed: first using the “phylosig” function from the *phytools* package, which estimates the joint lambda value across all amino acid data. The second method used the “fitContinuous” function from the R package *geiger* (Harmon et al., 2008); this method calculates a separate lambda value for each amino acid. The phylogeny used in the Pagel's lambda analyses was a maximum clade credibility tree developed from nuclear and mitochondrial sequence data (Burns, Hedin, & Shultz, 2012; Burns, Hedin, & Shultz, 2013) and pruned to represent the five taxa sampled in this study.

## 3 RESULTS

Eleven free amino acids were detected in the harvestmen accessory glands and penial sacs examined, with five amino acids making up 91% of the total free amino acid content: alanine, threonine, isoleucine, glutamine, and serine. In order to verify the suitability of frequentist statistics for downstream comparative analysis of nuptial gift profiles, we used two methods to calculate Pagel's lambda. Using the “phylosig” function (Revell, 2012) yielded a value of 0.0000661 (joint LnL = 9.93). Using the “fitContinuous” function (Harmon, Weir, Brock, Glor, & Challenger, 2008), we estimated λ = 0 for every amino acid variable, with a mean log‐likelihood of 16.63. With sufficient support for the lack of phylogenetic signal in nuptial gift content, we proceeded with frequentist analytics for comparison and contradistinction of amino acid profiles.

Eight of eleven amino acids identified were significantly different in average proportion for sacculate versus nonsacculate species according to an analysis of variance between the two morphological conditions (alanine: *F* ratio = 18.0283, *p* < 0.0001; threonine: *F* ratio = 21.1217, *p* < 0.0001; isoleucine: *F* ratio = 16.2977, *p* < 0.0001; serine: *F* ratio = 14.3914, *p* < 0.0003; glutamic acid: *F* ratio = 30.0792, *p* < 0.0001; valine: *F* ratio = 17.1605, *p* < 0.0001; phenylalanine: *F* ratio = 22.9569, *p* < 0.0001; tyrosine: *F* ratio = 20.2757, *p* < 0.0001). There was no significant difference in glutamine, glycine, or cysteine proportions between the two groups. Valine and tyrosine were only present in nonsacculate species. Sacculate species produced gifts with an average EAA proportion of 0.392 ± 0.021 (Md: 0.391, Q1: 0.359, Q3: 0.421). Nonsacculate species produced gifts with an average EAA proportion of 0.330 ± 0.022 (Md: 0.320, Q1: 0.286, Q3: 0.359). An ANOVA comparing the EAA content of sacculate and nonsacculate species yielded a *p*‐value <0.0001 with sacculate species gifts on average containing a 19% larger proportion of EAAs (*F* ratio = 16.9768, Prob. *F* < 0.0001). Using phylogenetic ANOVA, a similar and statistically significant pattern was also found (*F* = 8.045, Prob. Sim. *F* < 0.05). We did not find ethanol preservation level (Welch‐corrected *t* = 1.578, *p* = 0.1219), locality (Welch *t* = 0.2573, *p* = 0.8003) or use in prior mating trials (Welch *t* = 0.7784, *p* = 0.4459) to significantly affect EAA proportions within species.

**Figure 3:**
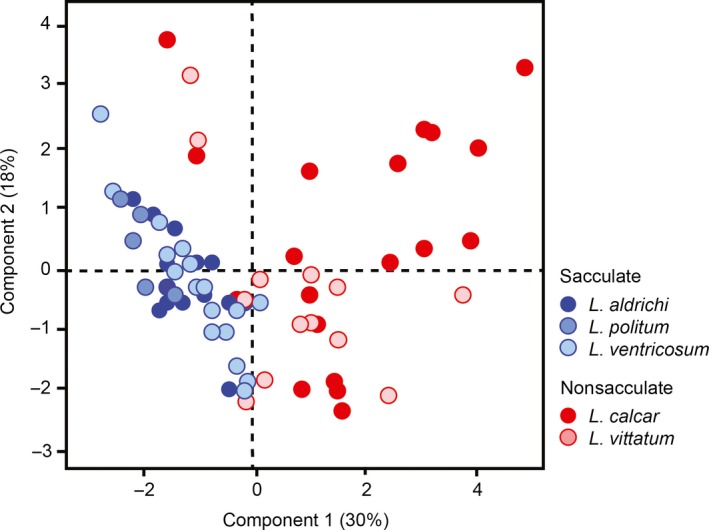
Principal components analysis of free amino acid content of nuptial gift illustrating differences between sacculate (blue) and nonsacculate (red) genital morphotypes. PC1 explains 30.2% of the variance in the data and is characterized by positive loading of alanine, valine, phenylalanine, glutamic acid, and tyrosine, and negative loading of isoleucine and threonine. PC2 explains 17.7% of the variance in the data and is characterized by positive loading of valine, glutamine, and phenylalanine, and negative loading of glycine, serine, and glutamic acid

Principal components analysis (PCA) on the free amino acid profiles of the harvestmen specimens yielded four PCs with eigenvalues exceeding 1, which collectively explain 70.6% of the total variation in amino acid composition. PC1 explains 30.2% of the variation and is characterized by positive loading of alanine, valine, phenylalanine, glutamic acid, and tyrosine, and negative loading of isoleucine and threonine. PC2 accounts for a further 17.7% of the variation and is characterized by positive loading of valine, glutamine, and phenylalanine, and negative loading of glycine, serine, and glutamic acid. PC3 explains 12.7% of the variation and is characterized by positive loading of glutamine and serine, and negative loading of alanine and cysteine. At last, PC4 accounts for 9.98% of the variation and is characterized by positive loading of serine and cysteine, and negative loading of alanine. The sacculate species scores largely overlap with each other but plot almost exclusively within the negative range of PC1, whereas the nonsacculate species plot on the positive half of PC1 (Figure 3). There are visible differences between the phenotypes in clustering; of the two nonsacculate species, *L. calcar* and *L. vittatum*,* L. vittatum* predominantly plots on the negative half of PC2 (excluding two outliers), whereas *L*. *calcar* is more variable across PC2 (Figure 3). Sacculate species cluster tighter, indicating less variation in the free amino acids of their nuptial secretions as compared to nonsacculate species (Figure 3).

## 4 DISCUSSION

Nuptial gift extractions from five species of leiobunine harvestmen confirmed the presence of free amino acids in varying combinations and concentrations. The primary contributing amino acids from all five species were alanine, threonine, isoleucine, glutamine, and serine, with secondary contributors composed of glycine, cysteine, glutamic acid, valine, phenylalanine, and tyrosine. This profile is very different from what has been observed in crickets, in which the most abundant free amino acids in spermatophores were glycine and proline (Warwick et al., 2009; Gershman et al., 2012; Jarrige, Body, Giron, Greenfield, & Goubault, 2015). One factor that could cause this difference in nuptial gift makeup is variation in sensory/gustatory factors; some literature reports that ability and perception of taste vary widely among taxa (Gordesky‐Gold, Rivers, Ahmed, & Breslin, 2008; Nelson et al., 2002). Furthermore, if different harvestman species have gustatory preferences for different amino acids, it could account for the differences we detect between species in the current study. Trophic positions and differences in diets between crickets and harvestmen must also be considered. The chief source of nutrition in crickets comes from plant material, whereas harvestmen of the genus *Leiobunum* mainly eat small insects and worms (Acosta & Machado, 2007). Gaur (2014) demonstrated that, on average, plants incorporate 5% more glycine, 3% more proline, and 5% less isoleucine into their nonmembrane proteins than invertebrates do. This pattern could account for some of the variations of free amino acid composition.

Within the genus *Leiobunum*, adjustments to the chemistry of the nuptial gift have occurred over the course of its evolutionary history; our data indicate that these adjustments are associated with transitions between sacculate and nonsacculate genital morphotypes (Figure 1). Specifically, we found a significantly greater proportion of essential amino acids in the nuptial gifts of sacculate species as compared to nonsacculate species. Because essential amino acids must be derived from the environment, our results are consistent with a scenario in which evolutionary transitions have occurred between putatively costly and valuable nuptial gifts in sacculate species to less valuable nuptial gifts in nonsacculate species. In addition, we find evidence for not only overall qualitative similarities of nuptial gift within reproductive morphotypes of harvestmen, but componential similarities as well. Principal components analysis revealed that chemical profiles did not cluster based on phylogenetic relationships, but rather by genital morphotype (Figure 3). For example, *L. ventricosum* and *L. aldrichi* (both sacculate) share almost the same multivariate space, but they are not each other's closest evolutionary relatives; *L. aldrichi* is more closely related to both of the nonsacculate species than it is to *L. ventricosum*. Similar to that, *L. vittatum* and *L. calcar* (both nonsacculate) overwhelmingly dominate the positive half of PC1, but the latter is more closely related to *L. politum* (sacculate; Figure 3).

Previous work using Bayesian‐likelihood estimation predicts that the sacculate condition is ancestral within *Leiobunum* and that the nonsacculate condition evolved twice, separately for each nonsacculate lineage (Burns et al., 2013; Figure 2). Another possible but less parsimonious transitional history requires that the common ancestor of the *L. vittatum, L. politum,* and *L. calcar* species groups lost penial sacs, which were then regained by the *L. politum* species group. Both situations involve two separate phenotypic transitions, but the latter is less likely because, in terms of development, it is easier to lose the genetic framework necessary to build a structure than it is to gain said framework (Dollo's Law; Marshall, Raff, & Raff, 1994). Presumably, the similarity in nonsacculate nuptial gifts is also the result of convergent evolution. We found that the nuptial gift of the nonsacculate species *L. vittatum* and *L. calcar* gifts is strikingly similar; for example, they both display novel acquisition of valine and tyrosine. We therefore hypothesize that the evolution of the nonsacculate condition promoted selection for a consistent, specific chemical composition of nuptial gift each time it arose.

Future work will be necessary to establish a functional context for nuptial gifts in these animals. Fowler‐Finn et al. (2014) described the mating behavior of *L. vittatum* in detail and observed that females often resist males’ advances—consistent with expectations of antagonistic interactions between sexes raised by the nonsacculate condition of males and the females’ sclerotized genital barriers. However, males sometimes struggle to end copulation; the authors suggest that this dynamic is consistent with females’ interest in prolonging access to nuptial gift. In order to test the sensory trap hypothesis, feeding trials are a logical next step; potential experiments might include incorporating amino acids into gels at differing concentrations and compositions and observing feeding behavior in order to confirm which amino acids are attractive in this group. In order to test the paternal investment hypothesis, future research would ideally include observation of the effects of nuptial feeding on female postcopulatory behavior, oviposition, and egg size and number. Radiolabeling studies could be carried out to see how females incorporate these materials into their body tissues.

The identification and classification of other compounds (e.g., proteins, other small molecules) in the nuptial gift extractions were outside of the scope of this study, but should be investigated further. Large‐scale “‐omic” analyses have received some utility in the study of nuptial gifts, particularly those that contain whole proteins or molecules with known toxicity; Pauchet et al. (2015) sequenced the proteome of a cricket *G. sigillatus* spermatophylax and the transcriptome of the male accessory glands that make these proteins (Pauchet et al., 2015). Harvestmen also synthesize unpalatable toxins (i.e., benzoquinones) which ward off natural predators and prevent microbial infections (Rocha et al., 2013). Toxic materials can be costly to produce and so would be expected to make good candidates for nuptial donation.

The leiobunine harvestmen provide an intriguing and tractable system for the study of nuptial gifts. Differing levels of sexual antagonism are linked to transitions in both genital morphology and, as we have found in the current study, chemical composition of nuptial gifts. Specifically, a more solicitous sexual dynamic is associated with a higher proportion of essential amino acids, and potentially, a more nutritious gift. Our study is the first to investigate the chemical composition of nuptial gifts in harvestmen and the first to investigate the evolution of nuptial gift chemistry in a phylogenetic framework in any group of animals. Our results point to many potential avenues for continued research, both within and across species.

## CONFLICT OF INTEREST

None declared.

## AUTHOR CONTRIBUTIONS

M.B. and S.L.B. conceived the project; all authors contributed to study design. P.C.K., M.B., and S.L.B. collected specimens. P.C.K. and M.B. performed dissections. P.C.K. and D.D.C. performed chemical analyses. P.C.K. and M.B. analyzed the data. All authors contributed to the preparation of the manuscript.

## DATA ACCESSIBILITY

Data and other files used in analyses are archived at Dryad Digital Repository: https://doi.org/10.5061/dryad.550nd48.
